# Development of Co-Based Amorphous Composite Coatings Synthesized by Laser Cladding for Neutron Shielding

**DOI:** 10.3390/ma14020279

**Published:** 2021-01-07

**Authors:** Xiaobin Liu, Jiazi Bi, Ziyang Meng, Yubin Ke, Ran Li, Tao Zhang

**Affiliations:** 1 Key Laboratory of Aerospace Materials and Performance (Ministry of Education), School of Materials Science and Engineering, Beihang University, Beijing 100191, China; 18801310752@163.com (X.L.); bijiazi@buaa.edu.cn (J.B.); yang96hou@163.com (Z.M.); zhangtao@buaa.edu.cn (T.Z.); 2 Spallation Neutron Source Science Center, Institute of High Energy Physics (IHEP), Chinese Academy of Sciences (CAS), Dongguan 523808, China; keyb@ihep.ac.cn

**Keywords:** metallic glass, laser cladding, composite coating, crack inhibition, neutron shielding

## Abstract

Advanced amorphous coatings consisting of Co-based metallic glasses with ultrahigh strength (6 GPa) and high microhardness (up to 17 GPa) can significantly improve the surface properties of matrix materials. However, the intrinsic brittleness of Co-based metallic glasses can lead to the initiation of microcracks caused by the inevitable generation of thermal stress during the laser cladding process, which severely limits the potential application. In this paper, the methods of increasing substrate temperature and fabricating composite coatings with the addition of toughened Fe powders were adopted to inhibit the generation of microcracks in the Co_55_Ta_10_B_35_ amorphous coatings. Moreover, neutron shielding performances of the cladding coatings with high B content were investigated with a wide range of neutron energy (wavelength: 0.15–0.85 nm). The results indicate that the fully amorphous coating and composite ones can be fabricated successfully. The increase in the substrate temperature and the addition of Fe powders can effectively inhibit the initiation and propagation of microcracks. The fully Co-based amorphous coating with high B content (35 at.%) can exhibit excellent neutron shielding performance. With the addition of Fe powders, the neutron shielding performance is reduced gradually due to the dilution effect of B in the composite cladding coatings, but the microcrack will be completely restrained.

## 1. Introduction

Amorphous alloys (also called “metallic glasses”) show special atomic arrangement structure in long-range disorder and short-range order without microstructural characteristics like grain boundary and phase segregation. As a result, amorphous alloys can exhibit novel physical and chemical properties, such as large elastic strain, high strength, high hardness, excellent corrosion and wear resistances and so on, compared with traditional crystalline alloys [1,2]. However, the limitations of glass-forming ability (GFA) and brittleness at room temperature for amorphous alloys seriously hinders their potential applications.

The development of two-dimensional coating is an effective way to overcome size limitations caused by GFA and take advantage of the excellent properties of amorphous alloys [3,4,5]. Through surface technology, fabrication of an amorphous coating on substrate material can greatly improve its surface properties. The prepared amorphous coating can not only inherit the advantages of high strength, high hardness and good wear and corrosion resistance for bulk metallic glasses, but also, for the most part, avoid the limitations of glass formation over the critical size. So far, many processing techniques, such as plasma spraying [6,7,8] and high-velocity oxygen fuel (HVOF) [9,10,11], cold spaying [12,13], and laser cladding [14,15,16], have been adopted to deposit amorphous coatings. Compared with other techniques, laser cladding exhibits the following merits: high-energy input density, low dilution rate of coating, fast heating and cooling rate [17,18,19], wide glass-forming conditions, good metallurgical bonding with substrate [20], accurate selection cladding, less material consumption, and cost-saving.

Since Yoshioka et al. prepared Ni-Cr-P-B amorphous coatings on the surface of mild steel by laser cladding in 1987 [21], a large amount of works have been done to develop Zr-, Ti-, Cu-, Fe-, and Ni-based amorphous coatings using the laser technique [14,19,22,23,24,25,26,27,28,29]. Due to the rapid heating and cooling characteristics of the laser cladding process, large thermal stress generates and leads to the initiation and propagation of microcracks in the prepared amorphous coatings, which is unfriendly to the formation of unbroken coatings with brittle amorphous alloys. Li et al. used multi-laser scanning to prepare Al-based amorphous alloys. First, a high-power laser was used to melt materials, and then a low-power laser was employed to remelt the coating so as to release thermal stress and prevent the crack [30]. Lu et al. used a similar three-laser scanning method to remove thermal stress in brittle Fe-based amorphous coating [3]. Ye et al. tried to prepare a crack-free Fe-based coating by heating the substrate to 422 °C. However, due to the precipitation of the Fe phase, the samples were not completely amorphous [31]. Jung et al. also used a heated substrate to eliminate cracks, and the results indicated that the method could be suitable for amorphous alloys with a large GFA [32]. So far, few studies have focused on the fabrication of Co-based amorphous ones with high hardness and high strength. Zhang et al. studied Co-based coatings prepared by laser cladding. Due to the influence of dilution and stir from the substrate, the coatings exhibit layered structure [33]. Shu et al. demonstrated fabrication of Co-based amorphous coatings with the high thermal stability (the supercooling liquid region is 44 K) by laser cladding [34]. The Co-based amorphous coatings also exhibit excellent wear resistance [35] and corrosion resistance [36].

In 2011, Wang et al. successfully developed Co-Ta-B amorphous alloys with compressive strength up to 6 GPa and specific strength of 650 Nm/g, which created a new maximum record of strength for metallic materials. Moreover, the alloys present high Vickers hardness up to 16 GPa and Young’s modulus up to 250 GPa [37]. However, the application is still limited by their low GFA and high brittleness.

In this study, a Co_55_Ta_10_B_35_ (at.%) amorphous alloy (developed by our group) was chosen with high strength of 6.0 GPa, high hardness of 16.1 GPa, high B concentration of 35 at.% and relatively high GFA (the critical diameter for glass formation: 1 mm) [37]. Amorphous powder of the Co-based alloy was produced firstly by gas atomization, and then the fully amorphous coating and the composite ones were deposited on mild steel substrate by laser cladding mixtures of the amorphous powders and pure Fe ones with different mass ratios using the optimal parameters. Because of the large neutron scattering cross-section of B element, which easily captures neutrons, the wide-wavelength-range neutron shielding performances of the resulting coatings were tested. The effects of increase in the substrate temperature and improvement of the mass ratio of ductile Fe powders to the Co-based amorphous ones on the initiation, propagation and suppression of crack in the resulting coatings were investigated.

## 2. Materials and Methods 

### 2.1. Preparation of Powders and Coatings

In order to prevent the composition deviation caused by the spattering of the B element, Co-B pre-alloy, according to the chemical nominal composition of Co_55_Ta_10_B_35_, was melted firstly by induction-melting under the protection of high-purity argon atmosphere. Then, the Co-B alloy was re-melted with Ta pieces by arc-melting in high-purity argon atmosphere, and the same ingot is melted repeatedly for 4 times to ensure homogeneity.

Co-based amorphous powders were manufactured through the high-pressure Ar-gas atomization method from the master alloy. To ensure the fluidity of the amorphous powders and eliminate the influence of particle size on the cladding results, the powders below 300 mesh were sieved out. The selected amorphous powders were dried for 24 h at 70 °C to remove all residual moisture before laser cladding. The cladding powders were pre-placed on the substrate to form a powder bed. A thicker pre-melting layer requires a higher energy input density and causes more aggressive spattering. However, a thin pre-melting layer will cause an upward movement of substrate element(s), leading to an increase in dilution rate and a decrease in coating quality and amorphous rate. An optimal thickness of ~80 μm per layer was adopted according to our abundant experiments.

Laser cladding process of Co_55_Ta_10_B_35_ amorphous coating was performed using an AHL-W180III laser equipment equipped with a 180 W Nd: YAG solid-state laser. The optimal cladding parameters were employed as follows: loading voltage of 150 V, scanning speed of 250 mm/min, pulse width of 0.5 ms, and pulse frequency of 15 Hz for the amorphous and composite coatings. The 1045 steel plates in the thickness of 3 mm were used as the substrate for the laser cladding process. The substrate surface was polished with 800 grit SiC sandpaper to remove oxide film and then cleaned in absolute ethyl alcohol under ultrasonic wave. The thickness of the powder layer is ~80 μm per layer, and 4 layers were applied to reduce the dilution of the substrate to the coatings.

In order to suppress the generation of cracks during laser cladding, the substrate is pre-heated and Fe powder is introduced. The heating of the substrate was carried out throughout the laser cladding process, which could relieve thermal stress of the cladding layer and thus suppress the generation of cracks. The reason for the adoption of Fe powder can be listed as follows: (1) It is considered that the remaining Fe phase in the coating, playing as an applied ductile phase, can absorb the residual stress during laser cladding, and reduce the possibility of cracks. (2) Co-Fe-Ta-B [38] itself is an amorphous system. With a lower metalloid element content, the alloy coating could exhibit the possibility to control cracks, and ensure the formation of amorphous systems to a certain degree. 

### 2.2. Characterization of Powders and Coatings

Morphologies of the powders and coatings were observed using a JSM-7500F scanning electron microscope. The morphology and alloy composition of the cladding layer were analyzed using JXA-8100 electron microprobe through backscattered electron dectection equipped with an energy dispersive spectrometer (EDS). Phase analysis of the powder and coating was performed by D/max 2500pc type X-ray diffraction (XRD), with Cu Kα radiation at a scanning speed of 2 °/min and in a scanning range of 20–80°. The microhardness of the coating was measured using a FM800 Vickers microhardness tester, with a load of 1.961 N for 10 s. Each sample was randomly measured at 10 points, and the average value was adopted as the final result.

### 2.3. Neutron Shielding Test

The neutron shielding tests of the cladding coatings were performed by Small Angle Neutron Scattering (SANS) constructed and operated by China Spallation Neutron Source (CSNS), and the incident neutron wavelength range is 0.15–0.85 nm. The sample size for the measurement of neutron shielding performance is limited strictly in the dimension of 50 mm × 50 mm × 3 mm for the steel-plate matrix and 50 mm × 50 mm × ~200 μm for the coating by controlling the mass of cladding powders. The transmitted neutron counts with and without samples were detected by a Monitor-3 (GEM type) while the incident neutron counts were recorded by Monitor-2 (He-3 type). The transmission rate was calculated by using the same equation in [39], for which the data were normalized by using the total incident neutron counts. The neutron shielding performance of the sample can be judged by measuring the wavelength-dependent transmission spectrum of neutrons through the samples, and the lower the transmission rate, the better the performance. 

## 3. Results and Discussion

### 3.1. Characterization of Powders and Coatings

**Figure 1:**
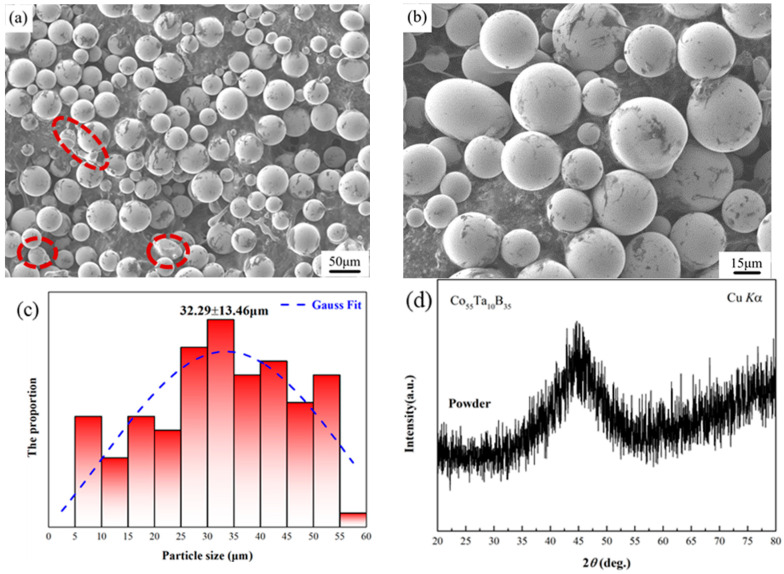
Microstructural characteristics of Co_55_Ta_10_B_35_ powders: (**a**,**b**) power morphologies at different magnifications; (**c**) the size distribution; (**d**) XRD pattern of Co_55_Ta_10_B_35_ amorphous powders.

Microscopic morphologies of Co_55_Ta_10_B_35_ powders are shown in Figure 1. The appearance of the amorphous powders is basically spherical or ellipsoidal with smooth surface, uniform particle size, and good compactness. A few lumpy irregular powder particles (marked by red dotted line frame) can be observed due to the contact between some liquid droplets and solidified powders under the action of high-pressure airflow resulting in the absence of spheroidization before the complete solidification to form solid powder. The distribution of particle size for the sieved powders is shown in Figure 1c. The average particle size of the powders is 32.29 μm. XRD pattern of the gas-atomized powders (Figure 1d) shows a single halo-like diffraction peak, indicating the fully amorphous powders of Co_55_Ta_10_B_35_ alloy can be fabricated through the high-pressure Ar-gas atomization method.

**Figure 2:**
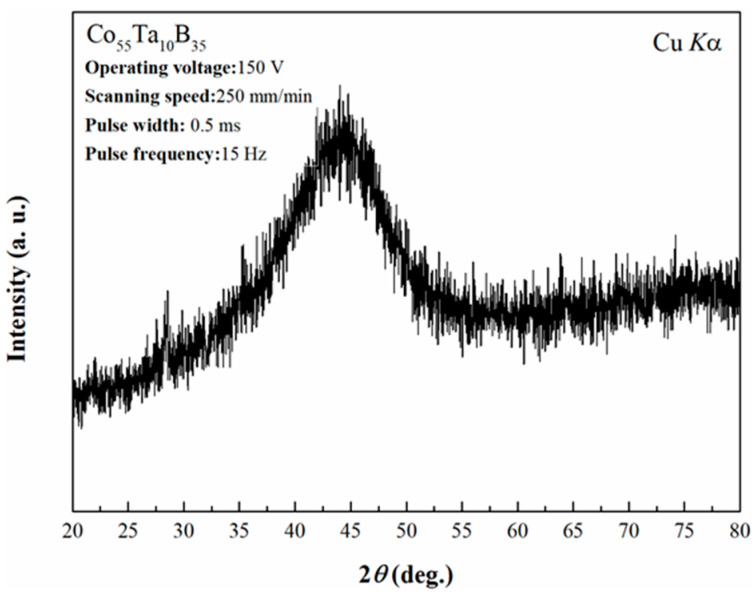
XRD pattern of Co_55_Ta_10_B_35_ amorphous coating prepared by laser cladding.

**Figure 3:**
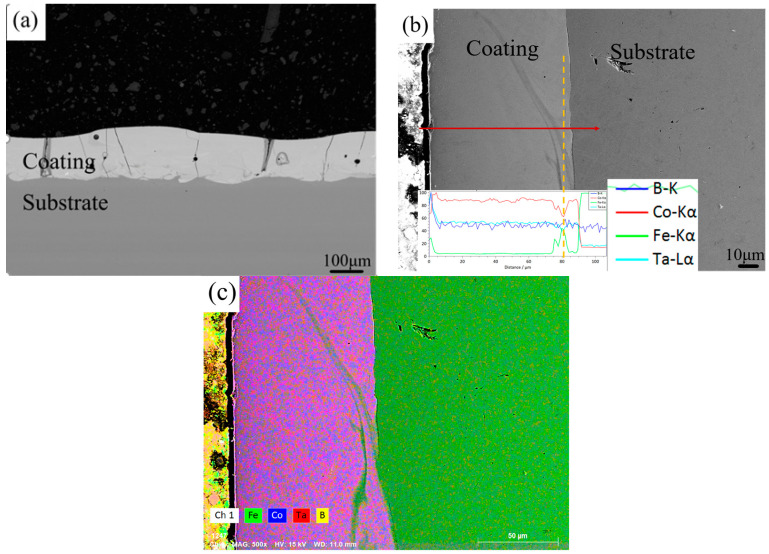
(**a**) Morphologies of Co_55_Ta_10_B_35_ amorphous coating in the cross-section view. (**b**) Elemental distribution from the coating to the steel matrix. (**c**) Mapping of elemental distribution.

The Co-based coating was fabricated firstly by laser cladding with the optical parameters (mentioned in Section 2.1) at room temperature. Figure 2 shows the XRD pattern of the coating. A single broad diffraction peak for the coating can be found like that of the powders, indicating that the fully amorphous coating can be produced. The cross-sectional morphology of the amorphous coating is shown in Figure 3. The interface between the coating and the substrate is well combined (Figure 3a), showing a good metallurgical bonding, but some microcracks can be also observed. Due to the low fracture toughness of Co-based amorphous alloys (*K*: 5 MPa·m^0.5^) [40] and high residual stress caused by rapid heating and cooling during the laser cladding process, some microcracks sprout along the vertical direction in the cladding layer, which will limit potential application of the Co-based amorphous coating. Figure 3b is the energy dispersive spectrum analysis of the amorphous sample from the cladding layer to the steel matrix. The elemental distribution in the coating is homogeneous and no obvious element segregation happens. The sharp increase in Fe content close to the matrix (marked by the red dashed line) may be due to the dilution effect of the steel substrate during laser cladding. The average Vickers hardness of the 1045 steel substrate was 1.5 GPa, while that of the amorphous coating measured increases of up to 14.9 GPa (about 10 times). The results indicate that Co_55_Ta_10_B_35_ amorphous coating prepared by laser cladding can significantly improve the surface hardness of the matrix. It was also found that the hardness of the Co-based amorphous coating produced by laser cladding is 1.2 GPa lower than that of the bulk amorphous alloy with the same composition prepared by copper mold casting. This could be attributed to more frozen free volume during the glass formation with a faster cooling rate by laser cladding than copper mold casting. The increase in free volume leads to the decrease in hardness value, which is consistent with previous results [41,42].

### 3.2. Investigation of Crack Control Methods

#### 3.2.1. Effect of Substrate Temperature on Coating

**Figure 4:**
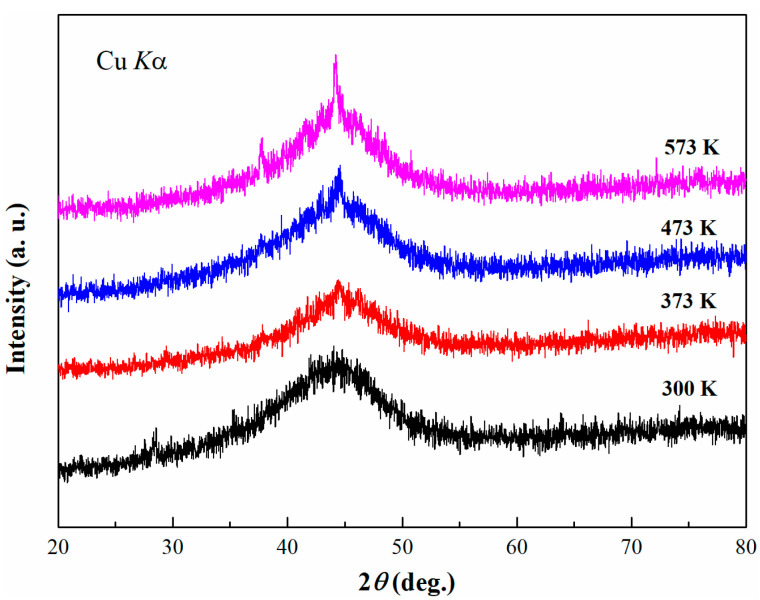
XRD patterns of Co_55_Ta_10_B_35_ coating prepared by laser cladding at different substrate temperatures.

Figure 4 shows XRD patterns of the Co-based amorphous coatings prepared at the different substrate temperatures. Some sharp diffraction peaks appear with the increase in the substrate temperature from 300 K to 573 K, suggesting that some crystalline phases occur during the laser cladding with high substrate temperature. Moreover, the stronger intensity of crystalline diffraction peaks, suggesting more crystallization, is accompanied with higher substrate temperature. Because the input energy density of laser remains a constant value, the cladding temperature within the heat-affected zone for the same material is basically the same. As the substrate temperature rises, the temperature gradient between the cladding layer and the substrate decreases. As a result, the cooling rate decreases in the heat-affected zone, responsible for a more remarkable crystallization behavior.

**Figure 5:**
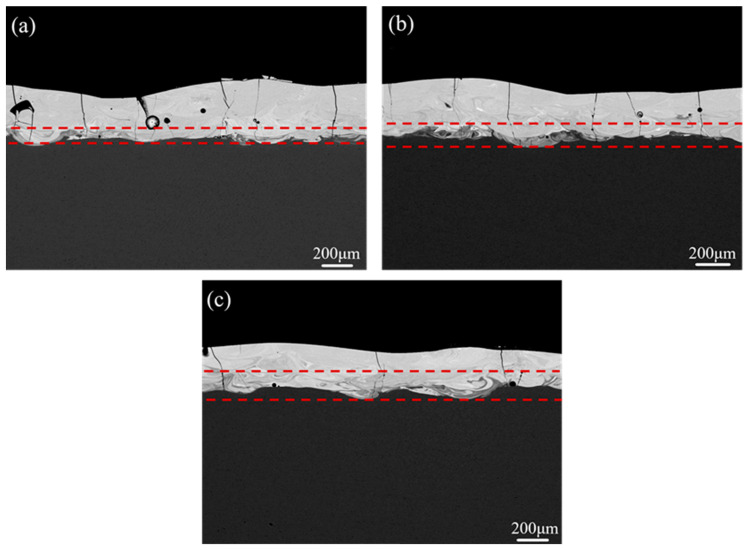
Backscattered electron images of Co_55_Ta_10_B_35_ amorphous coating prepared by laser cladding at different substrate temperatures: (**a**) 373 K; (**b**) 473 K; (**c**) 573 K.

**Figure 6:**
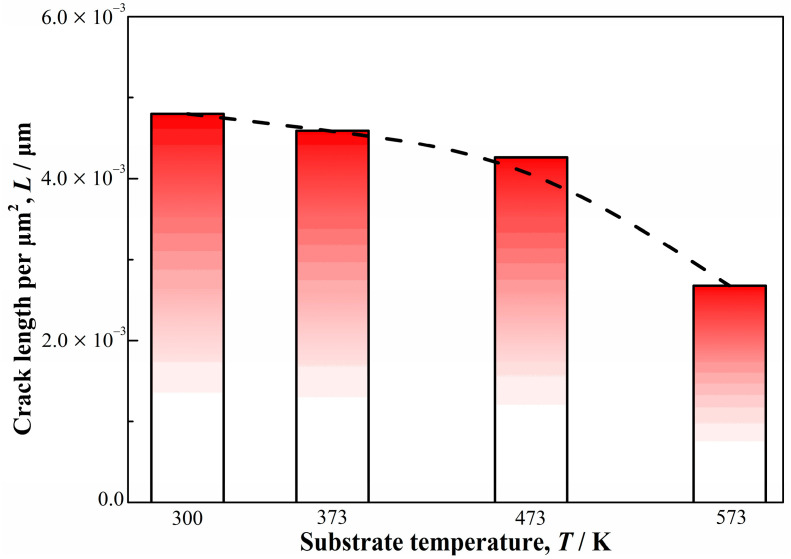
Cross-sectional cracking statistics of Co_55_Ta_10_B_35_ amorphous coating at different substrate temperatures.

The backscattered electron images of the cross section for Co_55_Ta_10_B_35_ amorphous coating at different substrate temperatures are shown in Figure 5. As the substrate temperature increases from 300 K to 573 K, the longitudinal cracks in the cladding layers decrease significantly, and the transition zone between the amorphous layer and the substrate became more obvious. The number of cracks along the interface direction in the coatings cladded at different temperatures was counted, as shown in Figure 6. Five consecutive SEM images for each specimen were analyzed for cracks. The cracks’ length per unit area (*L*) in the coatings decreases with the increasing of the substrate temperature. As the temperature raises up to 573 K, *L* is only half as much as that at 300 K. Benefiting from the decrease in the temperature gradient between the substrate and the coating caused by the increase in substrate temperature, thermal stress generated during the laser cladding process decreases, which plays a key role in the suppression of crack initiation. However, the low cooling rate caused by the increase in substrate temperature also improves the crystallization in the coating (Figure 4) and accelerates the dilution effect in the interface (Figure 5). The alloy composition in the coatings cladded at different temperatures was also analyzed by EDX, and the data are listed in Table 1. With the increase in the substrate temperature, the content of Fe element in the cladding layer increases slightly from 9.71% to 13.57% (due to the dilution effect of the laser), and the Co and Ta contents decrease accordingly, consistent with the above analysis. Therefore, the increase in substrate temperature can depress the generation of microcracks remarkably, while few microcracks still developed due to the crystallization and the dilution effects.

#### 3.2.2. Fabrication of Composite Coatings with the Introduction of Fe Powder

**Figure 7:**
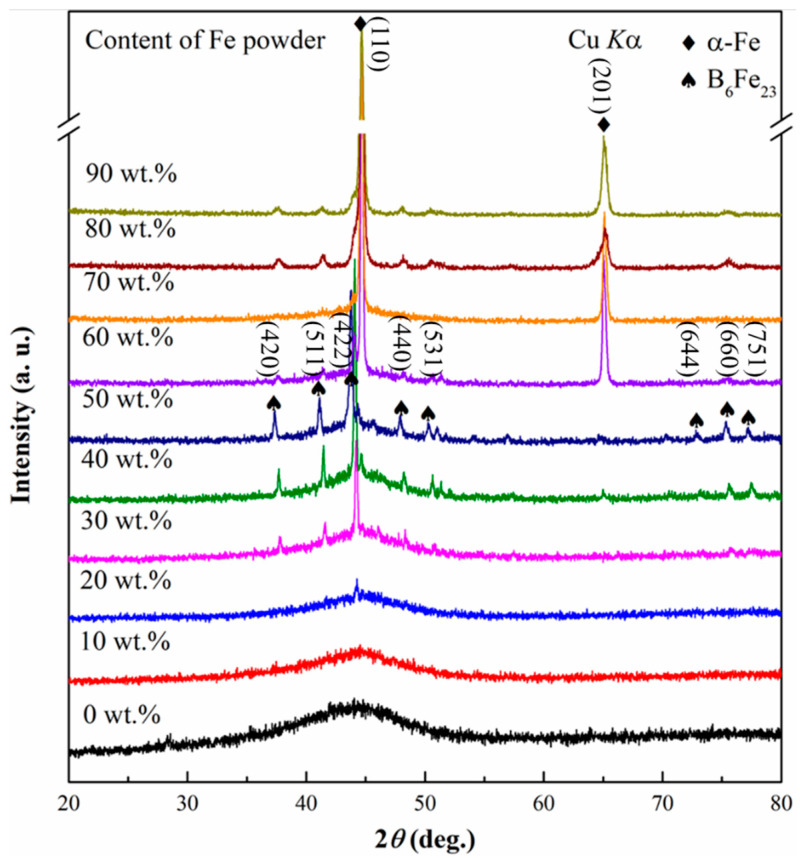
XRD patterns of the Co-based amorphous coating and the composite coatings produced by laser-cladding the mixtures of Co_55_Ta_10_B_35_ amorphous powder with different contents of Fe powder.

**Figure 8:**
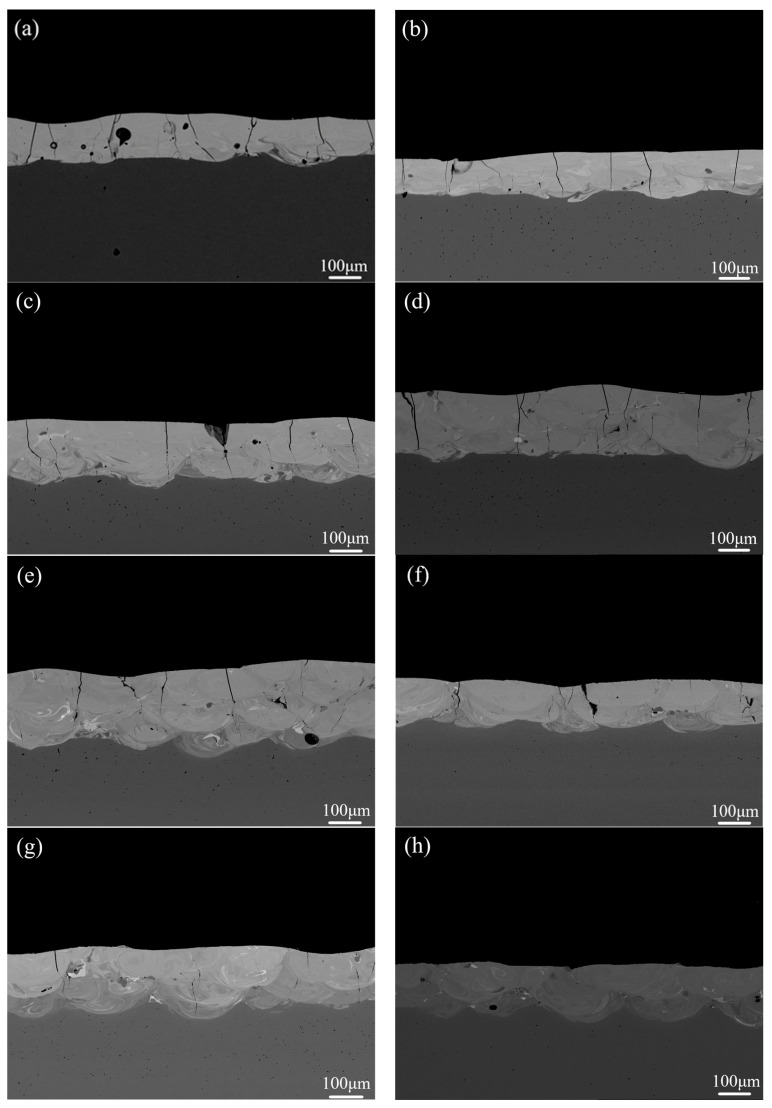

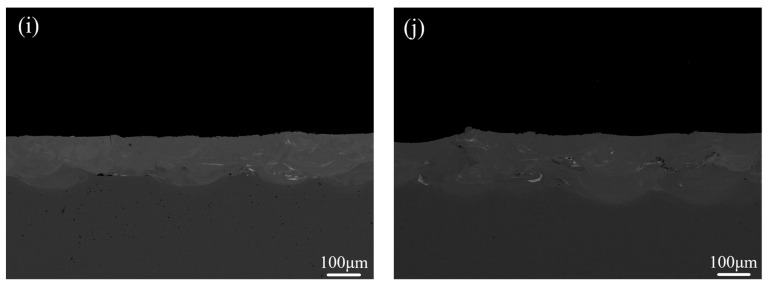
Cross-sectional morphology of Co_55_Ta_10_B_35_ composite coating prepared by adding Fe powder: (**a**) Fe-0 wt.%; (**b**) Fe-10 wt.%; (**c**) Fe-20 wt.%; (**d**) Fe-30 wt.%; (**e**) Fe-40 wt.%; (**f**) Fe-50 wt.%; (**g**) Fe-60 wt.%; (**h**) Fe-70 wt.%; (**i**) Fe-80 wt.%; (**j**) Fe-90 wt.%.

**Figure 9:**
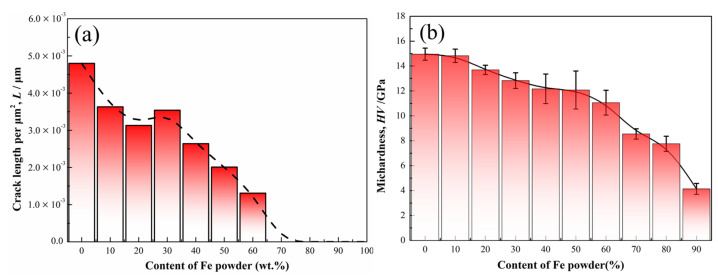
(**a**) Length statistics of cracks in cross section and (**b**) Vickers hardness of Co-based composite coatings prepared by laser cladding with the different mass contents of Fe powder.

In order to fabricate the crack-free coating, the mixtures of the Co-based amorphous powder and Fe powder in the different mass ratios were melted by laser cladding with the same laser parameters. Figure 7 shows XRD patterns of the composite coatings cladded from the mixtures with a different mass percent of Fe powder (i.e., 0 wt.%, 10 wt.%, 20 wt.%, 30 wt.%, 40 wt.%, 50 wt.%, 70 wt.%, 80 wt.%, and 90 wt.%). As the mass fraction of the added Fe powder is not more than 20 wt.%, the cladding layers mainly consist of amorphous phase, and only slight α-Fe precipitation phase can be found in Fe-20 wt.% sample. As the mass fraction of Fe powder remains at a higher level from 30 wt.% to 60 wt.%, the diffraction peaks of B_6_Fe_23_ intermediate phase can be found in the background of amorphous matrix. When the mass fraction of Fe powder increases to more than 60 wt.%, α-Fe phase can be mainly identified, with a slight B_6_Fe_23_ phase in the cladded samples. Morphologies of the amorphous coating and composite ones in the cross-sectional region are shown in Figure 8. With the increase in the mass fraction of Fe powder, microcracks in the cladding layers are gradually reduced. Combined with XRD results in Figure 7, the introduction of Fe powder can tune the chemical composition of Co-based amorphous alloys to a certain degree (low than 30 wt.%) and toughen the cladded amorphous layer. Further addition of Fe powder from 30 wt.% to 60 wt.% can form the composite coating consisting of amorphous phase and B_6_Fe_23_ phase with a better ability to inhibit the imitation of microcracks. With the gradual increase in Fe powder content, the improvement of the toughness and plasticity of the composite coatings is more obviously caused by the appearance of α-Fe phase. As the mass fraction of Fe powder increases up to 70%, no micro-crack can be observed in the cladding layer. *L* values of microcracks in the cross-sectional area for the coatings produced with different additions of Fe powder were also counted, and the results are shown in Figure 9a. The values of *L* decrease gradually with the increase in the content of Fe powder. As the mass ratio of Fe powder to Co-based amorphous powder reaches 70 wt.%, the crack-free composite coating can be fabricated successfully. 

With the increase in the amount of Fe powder added (Figure 9b), Vickers hardness of the composite coatings gradually decreases. As the mass fraction of added Fe powder is small (less than 20%), the coating shows amorphous structure. Thus, its hardness remains at a high level of ~15 GPa. As the content of Fe powder increases up to 30–60 wt.%, the hardness values of the coatings with the composite structure of amorphous phase and B_6_Fe_23_ phase still remain at a high level of 12–14 GPa. The error bars appear more obvious fluctuation with the increase in Fe powder content, caused by the aggravated precipitation for the intermediate phase with inhomogeneity in the composite coatings. When the added Fe powder reaches up to 70 wt.%, the hardness of the crack-free coating is still 8.55 GPa, about 6 times that of the matrix.

### 3.3. Neutron Shielding Performance Analysis

**Figure 10:**
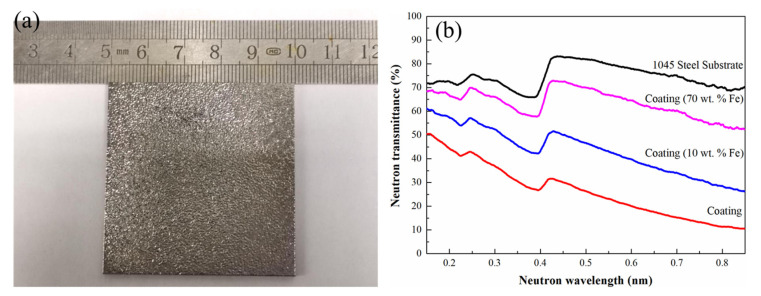
(**a**) Appearance of typical laser-cladded samples for neutron shield testing. (**b**) Neutron transmittance at different wavelengths for the coatings with the different additions of Fe powder. The background data for the 1045 steel substrate are also shown for comparation.

The appearance of the typical coating samples with a dimension of 50 mm × 50 mm for the neutron shielding measurement is shown in Figure 10a, demonstrating the successful fabrication of the large-area cladding layer. The neutron transmission rates of the selected samples of the amorphous coating, composite coating of Fe-10 wt.% and Fe-70 wt.%, and 1045 steel substrate in the wide range of neutron wavelengths are shown in Figure 10b. Due to the high neutron absorption cross-section of the B element, the sample with 200-μm-thick Co_55_Ta_10_B_35_ amorphous coating shows good shielding effect, i.e., less than 50% neutron transmission rate in a wide range of wavelengths of 0.15–0.85 nm. Compared with the 1045 steel substrate (above 70%), the neutron transmission rate of the amorphous coating with high B concentration remains at a low level in the full wavelength range measured. Especially, for neutrons with wavelength of 0.85 nm, the transmittance rate is only 10.5%, and the corresponding substrate material exhibits a transmission rate of 70.3%, which increases the absorption ability approximately 6 times. Considering the thin coatings of only ~200 μm, remarkable shielding effect is demonstrated in this material. The neutron shielding performances of the composite coatings of Fe-10 wt.% (fewer cracks) and Fe-70 wt.% (crack-free) were also tested. With the increasing of the addition of Fe powder, the neutron shielding performance of the cladding coatings decreases gradually, caused by the diluted concentration of B in the coatings, i.e., a weaker neutron absorption ability. However, compared with the substrate, the composite coating of Fe-70 wt.% still shows a significant improvement of neutron shielding performance. A series of dips can be observed, appearing at the positions of 0.21 nm and 0.42 nm in the transmission spectrum, which is attributed to the characteristic Bragg diffraction effect of the crystalline steel substrate. Moreover, the irradiated samples do not exhibit further radioactivity after the neutron shielding experiments for about 40 min. The experimental results indicate that the Co-based amorphous coating and its composite ones with high hardness can exhibit superior neutron shielding effects in the wide energy range, suitable for the external neutron shielding protection as coating materials.

## 4. Conclusions

In this study, a family of Co-based amorphous coating and its composite ones was fabricated by laser-cladding the mixing powders with different mass ratios of Co_55_Ta_10_B_35_ amorphous powder and Fe powder. By increasing the substrate temperature and introducing toughening element of Fe, the initiation and propagation of cracks can be obviously inhibited in the composite coatings, while partial crystallization occurred in the cladding layers with slight decreases in hardness. The resulting coatings with Fe powder content of less than 20 wt.% show completely amorphous structure with hardness of up to 14.9 GPa, about 10 times that of the substrate of 1045 mild steel. The Co-based amorphous coating and its composite ones exhibit excellent neutron shielding performances in the wide wavelength range from 0.15 nm to 0.85 nm compared to that of the 1045 steel substrate. 

## Figures and Tables

**Table 1:** Composition analysis of Co_55_Ta_10_B_35_ coating in cross section produced at different substrate temperatures.

Substrate Temperature	300 K	373 K	473 K	573 K
Co (at.%)	78.86	76.68	75.63	75.51
Ta (at.%)	11.43	11.01	10.93	10.92
Fe (at.%)	9.71	12.31	13.44	13.57

## Data Availability

Data sharing is not applicable to this article.
